# In Association with Other Risk Factors, Smoking Is the Main Predictor for Lower Transcutaneous Oxygen Pressure in Type 2 Diabetes

**DOI:** 10.3390/biomedicines12020381

**Published:** 2024-02-07

**Authors:** Tomislav Bulum, Neva Brkljačić, Angelika Tičinović Ivančić, Maja Čavlović, Ingrid Prkačin, Martina Tomić

**Affiliations:** 1 Vuk Vrhovac University Clinic for Diabetes, Endocrinology and Metabolic Diseases, Merkur University Hospital, 10000 Zagreb, Croatia; 2 School of Medicine, University of Zagreb, 10000 Zagreb, Croatia; 3 Zagreb Country Public Health Institute, 10430 Samobor, Croatia; 4 Department of Internal Medicine, Merkur University Hospital, 10000 Zagreb, Croatia

**Keywords:** smoking, type 2 diabetes, atherosclerosis, peripheral artery disease, transcutaneous oxygen pressure

## Abstract

Type 2 diabetes mellitus (T2DM) significantly increases the risk of peripheral artery disease (PAD), and diabetes is the leading cause of nontraumatic amputations. This study investigated the risk factors for transcutaneous oxygen pressure (TcPO2) in T2DM, a noninvasive method to quantify skin oxygenation and the underlying microvascular circulation. The study included 119 T2DM patients (91 male/28 female). TcPO2 measurements were conducted with the Tina TCM4 Series transcutaneous monitor (Radiometer, Copenhagen, Sweden) and skin electrodes. Patients with TcPO2 < 40 mmHg were younger (*p* = 0.001), had significantly higher systolic blood pressure (SBP) (*p* = 0.023), glycated hemoglobin (HbA_1_c) (*p* = 0.013), fasting plasma glucose (fPG) (*p* = 0.038), total cholesterol (*p* = 0.006), LDL cholesterol (*p* = 0.004), and had more frequent smoking habits (*p* = 0.001) than those with TcPO2 ≥ 40 mmHg. The main predictors for the TcPO2 value (R^2^ = 0.211) obtained via stepwise regression analysis were age, smoking, SBP, HbA_1_c, fPG, and total and LDL cholesterol. Among all the listed predictors, smoking, HbA_1_c, and LDL cholesterol were found to be the most significant, with negative parameter estimates of −3.051310 (*p* = 0.0007), −2.032018 (*p* = 0.0003), and −2.560353 (*p* = 0.0046). The results of our study suggest that in association with other risk factors, smoking is the main predictor for lower TcPO2 in T2DM.

## 1. Introduction

Diabetes is a chronic disease with harmful chronic complications that is still the leading cause of blindness in the adult working population, nontraumatic amputations, and chronic kidney disease. Peripheral artery disease (PAD), caused by atherosclerosis and aggravated with several risk factors, affects the lower extremities and increases the risk of cardiovascular disease and death [1,2,3,4,5]. Compared to non-diabetic individuals, diabetes is associated with a two- to fourfold higher risk of PAD, while the presence of diabetes worsens outcomes in patients with PAD [6,7,8,9]. The worst diabetes complication is diabetic foot developed because of diabetic neuropathy and/or PAD [10].

Patients with type 2 diabetes mellitus (T2DM) usually have metabolic syndrome with a set of metabolic disturbances, like obesity, dyslipidemia, hypertension, and arterial stiffness, that increase the risk of cardiovascular disease and the risk of PAD [11,12,13,14]. It should be noted that PAD in diabetic patients is different at biological and clinical levels, and the effects of good glycemic control on the regression of microvascular disease are not reflected in macrovascular disease in the short term. There is a bidirectional relationship between T2DM and PAD; one disease may play an underlying role in the pathophysiology of the other and vice versa [15,16,17]. The ankle–brachial pressure index (ABI) is a noninvasive method to detect the presence and severity of PAD widely used in clinical practice [18,19]. However, in patients with diabetes, ABI has limited reliability in the diagnosis of PAD because of medial artery calcification, and the toe–brachial index (TBI) is more often estimated than ABI [20]. These two methods have limitations when examining the microcirculation in patients with PAD. A transcutaneous oxygen pressure (TcPO2) measurement provides information about the supply and delivery of oxygen to the underlying microvascular circulation by recording the partial pressure of oxygen at the skin surface. It can be considered a metabolic test, while ABI and TBI are hemodynamic tests [21,22].

ABI and TBI, as the most widely used noninvasive methods to detect the presence and severity of PAD in clinical practice, have limitations when examining microcirculation. Since diabetes is strongly associated with both macrovascular and microvascular complications, this study aimed to investigate the risk factors for lower TcPO_2_ in T2DM, a metabolic test that provides information about microvascular circulation.

## 2. Materials and Methods

### 2.1. Study Design and Ethics Statement

This study was cross-sectional and conducted at the Department of Diabetes and Endocrinology and the Department of Cardiology. It included 119 patients with T2DM, referred by a diabetologist/neurologist/vascular surgeon due to suspicion of PAD. PAD has been diagnosed using the color Doppler ultrasound of the superficial femoral and other more distal arteries of both legs. A normal spectral arterial waveform was triphasic, while biphasic and monophasic waveforms of one or several arteries were considered PAD [23]. In those with PAD, ABI and TcPO2 measurements were performed. Patients with an ABI ≤ 1.3 were included in the study. The study was approved by the Hospital’s Ethics Committee (protocol number 04/38-299, approval date: 7 August 2019). All T2DM patients, before any study procedures, received oral and written information about the study protocol and finally signed the written informed consent.

### 2.2. Demographic Data and Clinical Characteristics

Age at the time of the study, gender, and diabetes duration were basic demographic data of patients included in the study. Weight in kilograms (kg) and height in centimeters (cm) were measured to calculate body mass index (BMI) by dividing weight by the square of height in meters (kg/m^2^). The waist circumference (WC) and hip circumference were measured with a tailor meter on standard places on bare skin to calculate the waist-to-hip ratio (WHR). A digital sphygmomanometer was used to measure systolic blood pressure (SBP) and diastolic blood pressure (DBP) in a sitting position after the period of a 10 min rest and expressed in mmHg. A smoker was defined as a person who had a history of smoking a minimum of 100 cigarettes during life with current smoking on some days or every day.

### 2.3. Markers of Glycemic Control and Lipid Metabolism

Fasting plasma glucose (fPG), serum lipids (total cholesterol, triglycerides, low-density lipoprotein (LDL) cholesterol, and high-density lipoprotein (HDL) cholesterol), and hemoglobin A_1_c were measured in the morning after an overnight fast. Postprandial plasma glucose (ppPG) was measured two hours after a meal. Glycated hemoglobin (HbA_1_c) was measured using an automated turbidimetric inhibition immunoassay (HbA_1_c Gen 3, Cobas Integra 400 Plus, Roche Diagnostic, Basel, Switzerland) and expressed in % according to the National Glycohemoglobin Standardization Program (NGSP). This method is traceable to the International Federation of Clinical Chemistry and Laboratory Medicine (IFCC) reference system. Standard enzymatic methods were used to determine serum lipids, fPG, and ppPG on an automated analyzer (Beckman Coulter AU680, Beckman Coulter, Inc., Brea, CA, USA).

### 2.4. Transcutaneous Oxygen Pressure Measurement

The sensor/electrode of the Tina TCM4 series transcutaneous monitor (Radiometer, Copenhagen, Sweden) was put on the skin, where it heated the underlying tissue to create local hyperemia (making the arteries dilate), intensify the blood perfusion, and increase the oxygen pressure. Electrodes were heated up to 45 °C and transmitted a temperature of about 43 °C on the skin, improving oxygenation of the capillary blood. The sensor received an electric current corresponding to the oxygen concentration in the capillary blood. The oxygen diffused according to pressure from the capillary blood via the vascular epidermis to the electrode placed on the skin surface. After application, the sensor required a 10 to 15 min warm period and needed calibration every four to eight hours. A site reading involved an average of 35 min. Sensors were placed over a homogeneous capillary bed without hair, skin defects, ulcers, and prominent veins. Electrodes were not placed over a bone, on previous operation sites, in scar tissue, or in severe edema because they may have given unreliable results. Patients were advised to avoid prior smoking or caffeine use. During the monitoring, patients were in the supine position.

### 2.5. Ankle–Brachial Index

To calculate the ABI, measurements of the SBP in the brachial, posterior tibial, and dorsalis pedis arteries were conducted. The ABI represents higher resting SBP at the ankle divided by the higher systolic brachial pressure. A handheld 5 or 10 mHz Doppler instrument (Sonotrax Pro ultrasonic pocket Doppler, Edan, San Diego, CA, USA) was used to note the systolic pressures. A standard blood pressure cuff was placed immediately above the ankle to obtain the most precise pressure measurements. During the ABI measurement, patients were in the supine position. Normal ABI values are between 0.9 and 1.4. Values < 0.9 are indicative of peripheral atherosclerosis, while values over 1.4 are indicative of vascular calcification.

### 2.6. Statistical Analysis

Statistical analysis was conducted, and the graphs were created using the statistical package Statistica™ 14.0.1 (TIBCO Software Inc., Palo Alto, CA, USA). In all analyses, a *p*-value of less than 0.05 was considered statistically significant. After testing the normality of data distribution with the Kolmogorov–Smirnov test, normally distributed continuous variables were expressed as mean ± SD and non-normally distributed variables as the median with range. All categorical variables were expressed as numbers and percentages. Differences between the two groups were for continuous data examined using parametric (*t*-test) or non-parametric tests (Mann–Whitney), depending on the data distribution, and for categorical data testing, the chi-squared test was used. The Spearman rank correlation test was used to evaluate the relationship between the studied variables. Differences in TcPO2 between the groups according to the smoking habit (no, yes) and HbA_1_c value (<8.0%, ≥8.0%) and their interactions were tested using the two-way analysis of variance (ANOVA). Stepwise regression was used to detect the main predictors of TcPO2.

## 3. Results

This study included 119 T2DM patients (91 male/28 female) with a mean age of 68.5 ± 7.8 years and a mean diabetes duration of 19.7 ± 9 years. All included patients had PAD. According to the Fontaine classification [23], 34 were asymptomatic (stage I), 43 had intermittent claudication after more than 200 m of pain-free walking (stage IIa), 22 had intermittent claudication after less than 200 m of walking (stage IIb), 1 had stage III (ischemic rest pain), and 19 had stage IV (18 neuropathic ischemic ulcers and 1 gangrene). Asymptomatic patients were declared those who did not have typical pain symptoms in the leg muscles provoked by the effort that passes at rest but had a pathological color Doppler ultrasound finding regardless of the finding of ABI. ABI’s median (min–max) in those patients was 0.81 (0.34–1.25). Furthermore, all our included patients had some stage of diabetic neuropathy, i.e., 12 had stage 1 (intermittent numbness and pain), 99 had stage 2 (persistent numbness and pain), and 8 had stage 3 (debilitating pain). Their mean/median values of TcPO2, basic characteristics, all analyzed risk factors, and ABI are shown in Table 1.

Regarding the TcPO2 value, patients were split into two groups: group 1 (TcPO2 ≥ 40 mmHg; *n* = 66) and group 2 (TcPO2 < 40 mmHg; *n* = 53) (Table 2). The two TcPO2 groups did not significantly differ in gender, diabetes duration, anthropometric parameters, BMI, WC and WHR, DBP, ppPG, HDL cholesterol, and triglycerides (*p* > 0.05). However, patients with TcPO2 < 40 mmHg were younger (*p* = 0.001) and had more frequent smoking habits (*p* = 0.001) than those with TcPO2 ≥ 40 mmHg. Furthermore, those with lower TcPO2 had significantly higher SBP (*p* = 0.023), HbA_1_c (*p* = 0.013), fPG (*p* = 0.038), total cholesterol (*p* = 0.006), and LDL cholesterol (*p* = 0.004), while they had a significantly lower ABI (*p* < 0.001) than those with higher TcPO2.

**Figure 1:**
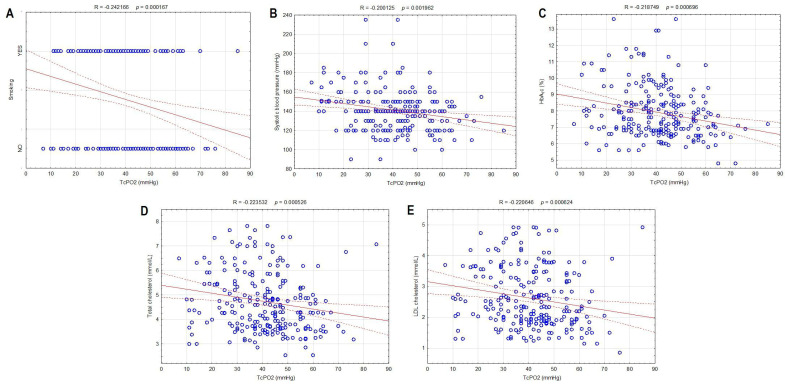
Correlations between transcutaneous oxygen pressure and smoking (**A**), systolic blood pressure (**B**), hemoglobin A_1_c (**C**), total cholesterol (**D**), and LDL cholesterol (**E**) in type 2 diabetic patients included in the study.

The correlation between TcPO2 and smoking and their relation to other risk factors and ABI are shown in Table 3. A significant negative correlation was found between the TcPO2 value and smoking (R = −0.242166, *p* = 0.000167) (Figure 1A). TcPO2 correlated significantly positively with age (R = 0.217315, *p* = 0.000757) while negatively with SBP (R = −0.200125, *p* = 0.001962) (Figure 1B), HbA_1_c (R = −0.218749, *p* = 0.000696) (Figure 1C), fPG (R = −0.153979, *p* = 0.017687), total cholesterol (R = −0.223532, *p* = 0.000526) (Figure 1D), and LDL cholesterol (R = −0.220646, *p* = 0.000624) (Figure 1E). In contrast, smoking related significantly negatively to age (R = −0.320846, *p* = 0.000000) and gender (m/f; R = −0.233076, *p* = 0.000287) but significantly positively to HbA_1_c (R = 0.294675, *p* = 0.000004) and ppPG (R = 0.137999, *p* = 0.033343) (Table 3). ABI was associated significantly positively with TcPO2 (R = 0.286434, *p* = 0.000039), while no significant correlation was observed between ABI and smoking (Table 3).

**Figure 2:**
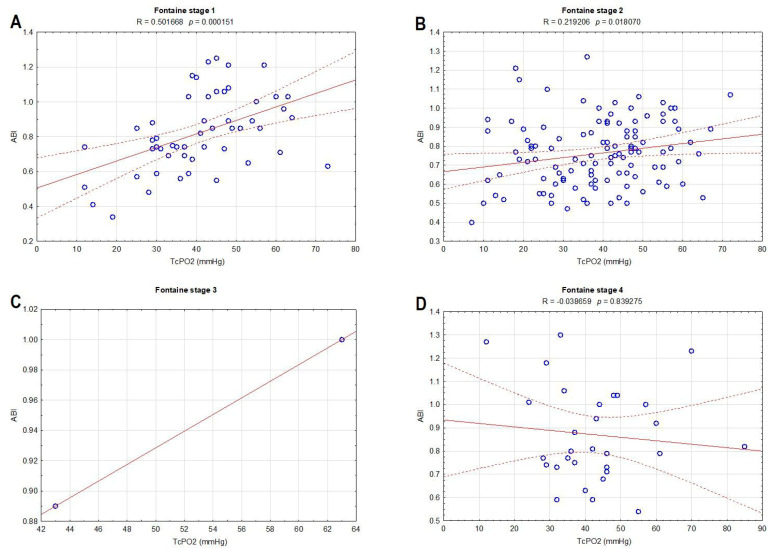
Correlations between the ankle–brachial index and transcutaneous oxygen pressure within different stages of the Fontaine scale: stage 1 (**A**), stage 2 (**B**), stage 3 (**C**), and stage 4 (**D**).

Figure 2 presents the correlation of ABI and TcPO2 within different stages of the Fontaine scale. The most significant association was observed in stages 1 (R = 0.501668, *p* = 0.000151) (Figure 2A) and 2 (R = 0.219206, *p* = 0.018070) (Figure 2B); in stage 3 (Figure 2C), there was a small number of patients to analyze, while in stage 4 (R = −0.038659, *p* = 0.839275) (Figure 2D), no significant relation between ABI and TcPO2 was observed.

**Figure 3:**
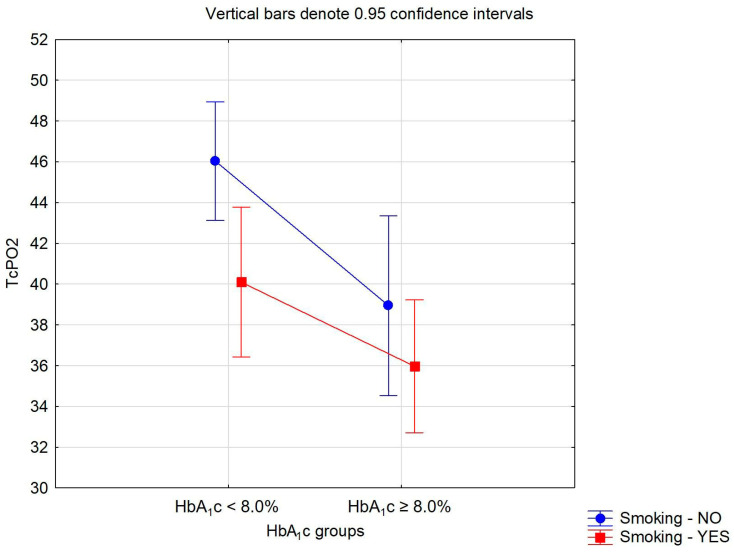
Differences in transcutaneous oxygen pressure (TcPO2) according to the smoking habit and glycated hemoglobin (HbA_1_c) value.

With a special emphasis on the two essential risk factors, smoking and HbA_1_c, Table 4 presents the differences in TcPO2 of T2DM divided into two groups according to their smoking habit (no, yes) and the level of HbA_1_c (<8.0%, ≥8.0%) tested via ANOVA with two main factors and their interaction. The statistically significant differences in TcPO2 were found according to the smoking habit (*p* = 0.016) and the level of HbA_1_c (*p* = 0.002), though the difference in TcPO2 observed according to the interaction between the groups of smoking and HbA_1_c was not significant (*p* = 0.419). The differences in TcPO2 found via ANOVA with two main factors and their interaction are graphically shown in Figure 3.

The main predictors for the TcPO2 value (R^2^ = 0.211) obtained via stepwise regression analysis were age, smoking, SBP, HbA_1_c, fPG, and total and LDL cholesterol (Table 5). Among all the listed predictors, smoking, HbA_1_c, and LDL cholesterol were found to be the most significant, with negative parameter estimates of −3.051310 (*p* = 0.0007), −2.032018 (*p* = 0.0003), and −2.560353 (*p* = 0.0046) influencing the TcPO2 value relative to a one-unit change of each and representing the negatively significant impact of the correlation between smoking and well-known risk factors on the reduction in TcPO2 value.

## 4. Discussion

The results of our study suggest that the main predictors for the lower TcPO2 value in T2DM obtained via stepwise regression analysis are age, smoking, SBP, HbA_1_c, fPG, total and LDL cholesterol. Among all the listed predictors, smoking, HbA_1_c, and LDL cholesterol were found to be the most significant. In contrast, no significant correlation was observed between ABI and smoking. TcPO2 is a metabolic test that measures oxygen pressure at the skin surface, while ABI and TBI, the preferred noninvasive indicators of PAD, have limitations when examining microcirculation in patients with PAD [21,22,23,24,25]. Diabetes mellitus facilitates the progression of medial artery calcification, which increases the values of systolic pressure at the ankle, and thus, T2DM patients diagnosed with PAD via a Doppler ultrasound might have a falsely normal ABI [24]. TcPO2 measurement should be conducted to assess the ischemia grade in those with arterial calcification where ABI and TBI are unreliable, in those without clinical symptoms like claudication and rest pain, and in those with diabetic foot complications [26,27,28]. This is following our results where no significant relation between ABI and TcPO2 was observed in stage 4 of the Fontaine classification (in our case, 18 patients with neuropathic ischemic ulcers and 1 with gangrene).

The mean age of T2DM patients in our study was 68.5 ± 7.8 years. It is well known that the prevalence of PAD increases with higher age. The prevalence of PAD in those over 50 years is up to 29% [29]. However, most elderly patients with PAD are asymptomatic because they walk slowly and cannot induce symptoms of PAD, such as rest pain and claudication [30,31]. Considering this, in older patients with T2DM and suspected PAD, TcPO2 might be a better diagnostic option in the assessment of PAD. In our study, older age was associated with better TCO2, which can be explained by the fact that older patients smoked less often and therefore had better TcPO2 results. The median blood pressure in our cohort was 140/80 mmHg, and SBP was slightly increased according to the latest diabetes guidelines [32]. A study that included over 4 million people between 30 and 90 years suggests that a 20 mmHg higher than the recommended SBP is associated with a 63% higher risk of PAD independent of sex or smoking status [33]. In addition, isolated systolic hypertension is the most prevalent form of hypertension in the population with PAD [34]. On the contrary, only those with lower DBP (<70 mm Hg) had a higher risk of PAD events in the lower extremities [35]. Intensive blood pressure control is effective and safe in patients with PAD [36]. Hypertension induces thickness of the smooth muscle cell layer of the media, fibrosis, remodeling of large arteries, and arterial stiffness, contributing to PAD [37,38,39].

In our study, HbA_1_c and fPG were significant predictors for the TcPO2 value. Advanced glycation end-products (AGE), promoted by hyperglycemia, accumulate in patients and may accelerate the progression of microvascular complications in diabetes [40]. In contrast, the relationship between hyperglycemia and the atherosclerosis of large arteries appears weaker [41]. AGE activates the expression of adhesion molecules on the surface and promotes the adhesion and entrance of monocytes/macrophages into the subendothelial space. AGE also modifies extracellular matrix molecules involved in developing atherosclerotic lesions [42]. Hyperglycemia also promotes proinflammatory responses via the activation of protein kinase C-beta and aldose reductase [43]. Hyperglycemia induces oxidative stress with superoxide overproduction in endothelial cells that activates several significant pathways involved in the pathogenesis of micro- and macrovascular complications of diabetes [44]. Finally, increased glucose uptake by vascular cells activates protein kinase C, an essential protein kinase mediating the cellular signaling pathway, and has several pro-atherogenic effects [45,46]. In ischemic conditions, hyperglycemia per se increases the susceptibility to limb necrosis [47].

Diabetic dyslipidemia is a known risk factor for PAD, and in T2DM nitrated lipoproteins, modified lipoproteins produced by the nitration of the tyrosyl residues of apolipoproteins by myeloperoxidase are also linked with cardiovascular disease [12]. The relationship between the level of LDL cholesterol, notably small dense LDL cholesterol, and the risk of atherosclerosis in T2DM is one of the most studied connections. In our study, total and LDL cholesterol were significant predictors for the TcPO2 value. In T2DM and insulin resistance, there is a predominance of small dense subclasses of LDL cholesterol that undergo several modifications, making them more atherogenic [48]. Small dense LDL cholesterol is associated with poor outcomes after vascular angioplasty in patients with PAD. Since our study included overweight T2DM, we can assume there was a predominance of small dense LDL particles in our patients [49,50]. In patients with type 1 diabetes with similar total and LDL cholesterol levels, as in our study, higher serum lipid levels were associated with lower TcPO2 [51].

The results of our study indicate that, in association with other risk factors, smoking is the main predictor for lower TcPO2 in T2DM. Smoking influences the distribution and composition of serum lipids, increases the total cholesterol content, promotes longer circulation of LDL in plasma and uptake in vessels, and accelerates the formation of plaques [52,53,54]. Cigarette smoke may induce the dysfunction of endothelial cells via decreased endothelial nitric oxide synthase activity and smooth muscle cells via several mechanisms [55,56]. Cigarette smoke directly damages cellular and sub-cellular structures via reactive oxygen and nitrogen species and the resulting oxidative stress [57]. Smoking causes vascular wall contraction by activating Rho kinase, promoting monocyte adhesion to endothelial cells and entering monocytes and macrophages into endothelium [58,59]. Nicotine from cigarette smoke causes migration, proliferation, apoptosis, phenotypic changes, and the contraction of smooth muscle cells in the arterial wall [60]. Smoking has a direct effect on the TcPO2 value because smoking increases carboxyhemoglobin and reduces oxyhemoglobin, therefore decreasing the TcPO2 value as well. However, a study that included 129 patients with PAD found that only the presence of diabetes adversely affected TcPO2 and clinical disease severity but not smoking [61]. Croatia has one of the highest smoking rates in Europe despite various anti-smoking campaigns. According to 2020 study data, the prevalence of smoking in Croatia was 36%, the third-highest tobacco prevalence in Europe [62].

Some limitations of the present study should be addressed. First, the cross-sectional design of our study limited the ability to infer a causal relation between TcPO2 and risk for the progression of PAD. Second, our study was a single hospital-based study; therefore, selection bias is likely. Third, this cohort had little racial/ethnic diversity, and our data would be primarily relevant to a white European population. Fourth, in patients with diabetes, TBI is more often estimated than ABI. Finally, a major limitation of the study is that smoking is recorded as a dichotomous variable, whereas it should be treated as a continuous one, given its effects vary significantly with the dosage consumed.

## 5. Conclusions

Our results suggest that, in association with other risk factors, smoking is the main predictor for lower TcPO2 in T2DM. No significant correlation was observed between ABI and smoking, and no significant relation between ABI and TcPO2 was observed in patients in the worst stage of the Fontaine classification, indicating that the TcPO2 measurement might be the preferable option to assess the ischemia grade in those with diabetic foot complications. In our everyday clinical practice, besides the optimal control of systemic risk factors, we need to be much more aware of the harmful effects of smoking, and patients with T2DM and PAD should be strongly advised to stop smoking.

## Figures and Tables

**Table 1:** Transcutaneous oxygen pressure, basic characteristics, risk factors, and ankle–brachial index of all type 2 diabetic patients (*n* = 119) included in the study.

	All Patients Included in the Study (*n* = 119)
TcPO2 (mmHg)	40.85 ± 13.97
Smoking (no/yes) (%)	50.4/49.6
BMI (kg/m^2^)	29.39 ± 4.88
WC (cm)	106.2 ± 12.5
WHR	0.99 ± 0.07
SBP (mmHg)	140 (90–235)
DBP (mmHg)	80 (41–115)
HbA_1_c (%)	7.90 ± 1.62
fPG (mmol/L)	8.16 ± 2.72
ppPG (mmol/L)	10.78 ± 3.54
Total cholesterol (mmol/L)	4.5 (2.5–9.2)
HDL cholesterol (mmol/L)	1.2 (0.4–2.3)
LDL cholesterol (mmol/L)	2.4 (0.3–4.9)
Triglycerides (mmol/L)	1.6 (0.6–9.5)
ABI	0.8 (0.3–1.3)

Legend: values are means ± SD, percentages, or median (min–max). BMI indicates body mass index; WC, waist circumference; WHR, waist-to-hip ratio; SBP, systolic blood pressure; DBP, diastolic blood pressure; HbA_1_c, glycated hemoglobin; fPG, fasting plasma glucose; ppPG, postprandial plasma glucose; HDL, high-density lipoprotein cholesterol; LDL, low-density lipoprotein cholesterol; ABI, ankle–brachial index.

**Table 2:** Transcutaneous oxygen pressure, risk factors, and ankle–brachial index of type 2 diabetic patients (*n* = 119) divided into two groups according to the transcutaneous oxygen pressure.

	TcPO2 ≥ 40 (*n* = 66)	TcPO2 < 40 (*n* = 53)	t^a^ χ^b^ Z^c^	*p*
TcPO2 (mmHg)	50.34 ± 8.85	29.12 ± 9.49	17.767 ^a^	<0.001
Age (years)	70.00 ± 7.45	66.75 ± 7.89	3.248 ^a^	0.001
Smoking (no/yes) (%)	59.5/40.5	38.7/61.3	10.201 ^b^	0.001
SBP (mmHg)	140 (90–230)	148 (100–235)	−2.267 ^c^	0.023
HbA_1_c (%)	7.67 ± 1.58	8.20 ± 1.64	−2.517 ^a^	0.013
fPG (mmol/L)	7.85 ± 2.14	8.58 ± 3.25	−2.082 ^a^	0.038
Total cholesterol (mmol/L)	4.3 (2.5–9.1)	4.8 (2.9–9.2)	−2.722 ^c^	0.006
LDL cholesterol (mmol/L)	2.2 (0.3–4.7)	2.6 (1.2–4.9)	−2.848 ^c^	0.004
ABI	0.8 (0.5–1.2)	0.7 (0.3–1.3)	3.539 ^c^	<0.001

Legend: values are means ± SD, percentages, or medians (min–max). t^a^ represents *t*-test, *χ*^b^ represents chi-square test, Z^c^ represents Mann–Whitney test, *p* represents comparison between patients with different level of TcPO2. SBP indicates systolic blood pressure; HbA_1_c, glycated hemoglobin; LDL, low-density lipoprotein cholesterol; ABI, ankle–brachial index.

**Table 3:** Correlations between transcutaneous oxygen pressure, smoking, the other risk factors, and ankle–brachial index in type 2 diabetic patients included in the study.

	TcPO2	Smoking
Smoking	−0.242 **	1.000
Age	0.217 **	−0.321 **
Gender (m/f)	−0.067	−0.233 **
SBP	−0.200 *	0.011
HbA_1_c	−0.219 **	0.295 **
fPG	−0.154 *	0.051
ppPG	−0.032	0.138 *
Total cholesterol	−0.224 **	0.096
LDL cholesterol	−0.129 *	0.059
ABI	0.286 **	−0.006

Legend: values are Spearman R-values. ** represents *p* < 0.001, * *p* < 0.05. SBP indicates systolic blood pressure; HbA_1_c, glycated hemoglobin; fPG, fasting plasma glucose; ppPG, postprandial plasma glucose; HDL, high-density lipoprotein cholesterol; LDL, low-density lipoprotein cholesterol; ABI, ankle–brachial index.

**Table 4:** Results of two-way ANOVA for the differences between transcutaneous oxygen pressure according to the smoking habit, hemoglobin A_1_c, and their interaction.

		TcPO2	
	df	F	*p*
Smoking	1	5.932	0.016
HbA_1_c gr.	1	9.394	0.002
Smoking and HbA_1_c gr.	1	0.656	0.419

Legend: TcPO2 indicates transcutaneous oxygen pressure; HbA_1_c, glycated hemoglobin.

**Table 5:** Results of stepwise regression analysis for the transcutaneous oxygen pressure as a dependent variable.

Variable	Estimate	Standard Error	F	*p*	Adjusted R^2^	R^2^
Age	0.355	13.716	9.65	0.0021	0.0353	0.211
Smoking	−3.051	13.656	11.83	0.0007	0.0439	
SBP	−0.145	13.645	12.21	0.0006	0.0454	
HbA_1_c	−2.032	13.598	13.93	0.0003	0.0519	
fPG	−1.109	13.665	11.46	0.0008	0.0424	
Total cholesterol	−1.959	13.771	7.71	0.0059	0.0277	
LDL cholesterol	−2.560	13.757	8.19	0.0046	0.0296	

Legend: TcPO2 indicates transcutaneous oxygen pressure; SBP, systolic blood pressure; HbA1c, glycated hemoglobin; fPG, fasting plasma glucose; LDL, low-density lipoprotein cholesterol.

## Data Availability

The data presented in this study are available on a specific request from the corresponding author.
